# Pandemic H1N1 influenza virus triggers a strong T helper cell response in human nasopharynx-associated lymphoid tissues

**DOI:** 10.1016/j.sjbs.2024.103941

**Published:** 2024-01-28

**Authors:** Waleed H. Mahallawi, Omar F. Khabour

**Affiliations:** a Clinical Laboratory Sciences Department, College of Applied Medical Sciences, Taibah University, Madinah, Saudi Arabia; b Department of Medical Laboratory Sciences, Faculty of Applied Medical Sciences, Jordan University of Science and Technology, Irbid, Jordan

**Keywords:** NALT, Mucosal vaccine, pH1N1, CD4^+^ T cells, Cross-reactive immunity

## Abstract

The pH1N1 belongs to influenza A family that is sometimes transmitted to humans via contact with pigs. Human tonsillar immune cells are widely used as *in vitro* models to study responses to influenza viruses. In the current study, human memory (M) and naïve (N) T cells responses in mononuclear cells of tonsil (TMCs) and peripheral blood (PBMCs) were stimulated by pH1N1/sH1N1, and then stained for estimation of T cells proliferation index. Individuals with an anti-pH1N1 hemagglutination (HA) inhibition (HAI) titer of forty or greater exhibited stronger HA-specific M-CD4^+^ T cells responses to pH1N1 in TMCs/PBMCs than those with an HAI titer of less than forty (P < 0.01). In addition, a positive correlation was observed between proliferation indices of M-CD4^+^ T cells induced by exposure to sH1N1/pH1N1 (p < 0.01). Moreover, a strong correlation (p < 0.001) was detected between subjects’ age and their HA-specific M-CD4^+^ T cells induced by pH1N1 exposure, indicating that this response was age-dependent. Finally, stimulation of TMCs with pH1N1-HA resulted in a significant M−CD8^+^ T cells response (p < 0.05). In conclusion, pH1N1 HA elicits a strong M-CD4^+^ T cells response in TMCs. Additionally, this response correlates with the response to sH1N1 suggesting cross-reactivity in T cells epitopes directed against HAs of both viral strains.

## 1 Introduction

The mucosal surface and local immune system lining the upper respiratory tract are essential for mediating immune responses and defense against influenza infections (Dotiwala and Upadhyay, 2023, Sandor et al., 2021). Numerous studies have recently employed the nasopharyngeal associated lymphoid tissues (NALT) model in respiratory infection research (Casadei & Salinas, 2019). Illustrative examples of the use of this model include SARS Co V-2 viral antigens and the tuberculosis vaccines (Aljeraisi et al., 2023, Altorki et al., 2023, Mahallawi and Aljeraisi, 2021b). The use of this model has facilitated advanced research on respiratory pathogen testing and intranasal vaccine development (Kurono, 2022, Pacini et al., 2023).

Helper CD4^+^ T cells are abundant in tonsillar tissue and are indispensable for the development of humoral immunity and the synthesis of influenza antibody via B lymphocytes (Lartey et al., 2020; B. O. Lee et al., 2005, Passàli et al., 2003, Sircy et al., 2023). One study reported an elevation in influenza antibody-secreting cells among individuals who received influenza vaccines, and the translocation of effector T cells toward the peripheral mucosa (Eriksson et al., 2003). Thus, influenza vaccine causes the recall of M-CD4^+^ T cells and enhances their ability to develop into a defense system against influenza. In influenza-infected rodents, M-CD4^+^ T cells are activated, proliferated, and then translocated to the infected lungs (Sant et al., 2018). More research is needed on vaccine-induced immunity, including neutralizing activity, which predicts immune protection from infectious agents (Kaufmann et al., 2017).

The current study examined the responses of memory and naive CD4^+^ T cells of TMCs to the 2009 pH1N1 virus and correlated the responses with the HAI serum antibodies of the subjects. The results will enhance our understanding of the immune response to H1N1 infection.

## 2 Materials and methods

### 2.1 Study participants

The tonsils were collected from forty patients (aged 1–38 years) who underwent tonsillectomy. Exclusion criteria were those patients who had influenza vaccines and patients with a compromised immune system. The Research Ethics Board of the Faculty of Applied Medical Sciences reviewed and approved the study protocol (IRB: 2023-MLT-012). Prior to their participation in this research, subjects signed a consent form after fully describing the study procedures as required by IRB.

### 2.2 Recombinant hemagglutinins (HA)

Both pH1N1 and sH1N1 HA proteins (A-California-04–2009, and A-Brisbane-59–2007 respectively) were obtained from the “National Institute for Biological Standards and Control”.

### 2.3 Tonsillar mononuclear cells (TMCs) isolation

The procedure for organizing cell suspensions was adopted from a previous study (Mahallawi & Aljeraisi, 2021a). Briefly, tonsil samples were pulverized before being transferred to a Petri dish. This was done to facilitate the discharge of TMCs from tissues into RPMI 1640 culture media (Sigma Aldrich). The cell suspension was introduced via a nylon mesh. Ficoll-Paque isolation of TMCs was performed (GE Healthcare, UK) using gradient centrifugation for 30 min at 400g. TMCs cells were counted in RPMI media and maintained at approximately 4 × 10^6^ cells/mL.

### 2.4 Assay for Haemagglutination inhibition (HAI)

HAI assay was conducted at the Colindale Microbiology Services (UK), according to established protocol (Kaufmann et al., 2017, Temperton et al., 2007). The virus strains used contained the following. For the pandemic H1N1 virus, NIBRG 122 virus is a reassortant organized from A/England/195/2009 (H1N1v), the prototype UK strain antigenically as well as genetically meticulously associated to A/California/4/2009; for the seasonal H1N1 virus, the A/H1N1/Brisbane/59/2007 strain was utilized. Briefly, 25 μL of a twofold serial dilution series from 1:8 to 1:1024 of RDE-treated serum samples were incubated for an hour at room temperature with an equal volume of the NIBRG-122 virus dilution, followed by the addition of 25 μL of the erythrocyte suspension.

### 2.5 Preparing M-CD4^+^ T cells

CD45RA^+^-depleted TMCs were used to study the response of M-CD4^+^ T cells to HA. CD4^+^ T cells proliferation was assessed using the carboxy fluorescein succinimidyl ester (CFSE) staining method (Valor et al., 2012). After TMCs were dissociated, naïve-T cells were selected using magnetic beads. CD4^+^ cells were enriched using the CD4 MultiSort Kit as described by the manufacturer (Miltenyi Biotec, UK).

### 2.6 Measurement of cell proliferation via flow cytometry

Proliferation of T cells and CFSE staining were performed as described (Hunt et al., 2016). Cells were exposed to various influenza antigens and proteins for stimulation. Next, cells were extracted in a 0.02 % solution consisting of bovine serum albumin (BSA)/phosphate buffer saline (Sigma-Aldrich). After washing, the cellular suspension was centrifuged, and the pellet was mixed with 50 µL BSA (0.02 %). For cell staining, mouse antibodies that detect CD4/PE/Cy5 and CD8/PE (BD) were used and applied for thirty minutes at 4 °C. The cells were then washed twice with BSA. After incubation, the pellet was centrifuged at 400g for eight minutes and analyzed after resuspension in BSA via flow cytometry (Becton Dickinson). Data were acquired using CellQuest software and analyzed using WinMDI 2.9.

### 2.7 Staining with carboxyfluorescein succinimidyl ester (CFSE)

The staining of TMCs using CFSE was achieved as described elsewhere (Valor et al., 2012). In brief, a working solution was made by mixing 5 µL of 5 mM CFSE into 10 mL of phosphate buffer saline. Cells were then incubated in 3 mL of working solution at 37 °C with 5 % CO2 for eight minutes. Then, 10 mL of ice-cold medium were added to the incubated cells. The mixture was then centrifuged for 10 min at 400 xg. Next, the cell pellet was reconstituted in 2 mL of RPMI. To induce cell proliferation, pN1H1 and sN1H1 were introduced into suspended cells at 37 °C/5% CO2 for 96 h. A flow cytometer was used to measure T cell proliferation (Becton Dickinson). Data were acquired using CellQuest software and analyzed using WinMDI 2.9.

### 2.8 Statistical analysis

Statistical and graphical analysis were achieved using “GraphPad Prism” (version 6) software. The *t*-test was used to compare experimental groups. Correlation analysis was employed using Pearson's correlation (r).

## 3 Result

### 3.1 Response of HA M-CD4^+^T cells to pH1N1

A Haemagglutination inhibition (HAI) assay was performed in patients’ serum using pH1N1 antibody. A positive HAI titer was defined as 40 or higher. The response of HA-specific M-CD4^+^ T cells of TMCs to pH1N1 was investigated. Individuals with a HAI titer of 40 or more had substantially higher CD4^+^ T cell proliferative response (p < 0.01) than those with a HAI titer of less than 40 (Fig. 1a). PBMCs exhibited a response like that of TMCs (Fig. 1b). However, the M-CD4^+^ T cells responses were more pronounced in TMCs than PBMCs.

**Fig. 1a:**
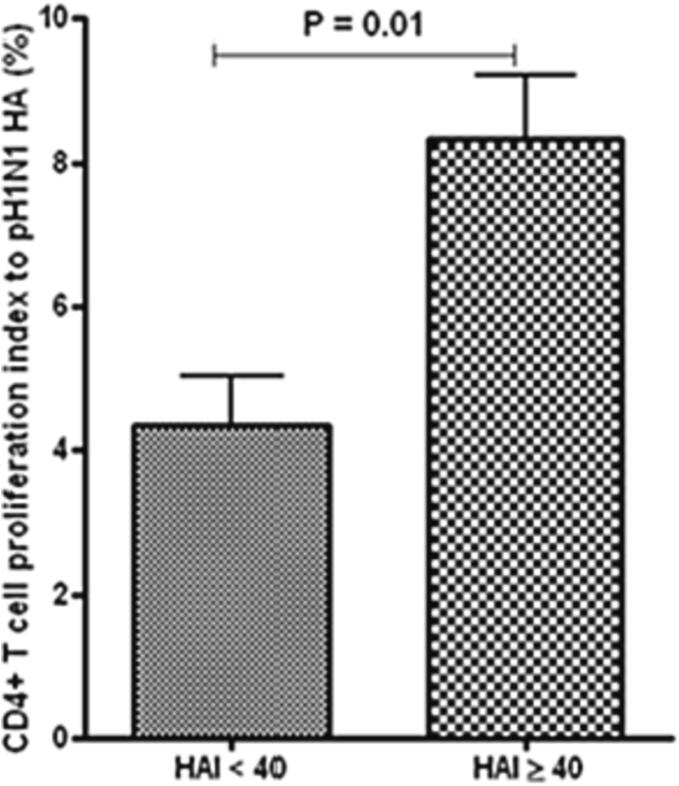
M-C**D4**^+^**T cell response in TMCs by pH1N1.** The M-CD4^+^T cell response in TMCs by pH1N1 was greater in individuals with an HAI titer of forty or greater than in those with an HAI of less than forty (n = 40 per group).

**Fig. 1b:**
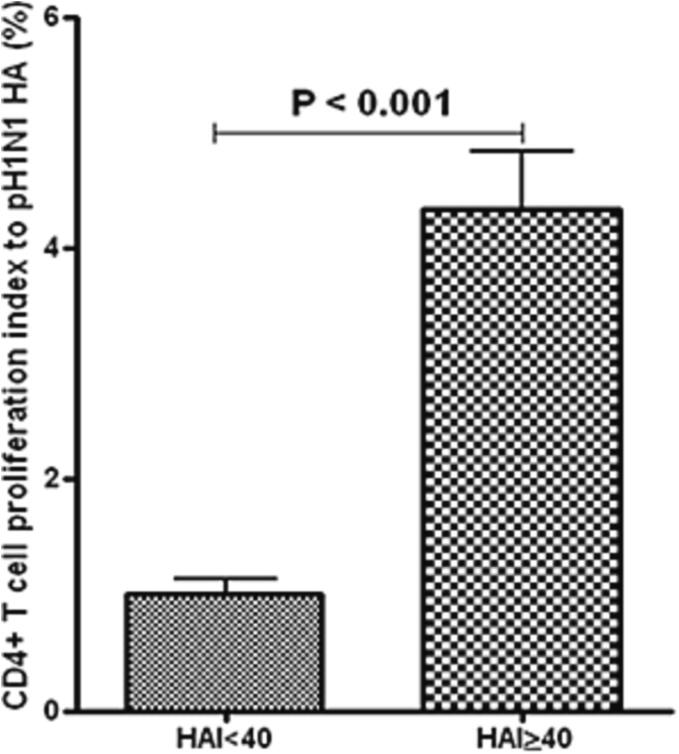
M-C**D4**^+^**T cell response in PBMCs by pH1N1.** The M-CD4^+^T cell response in PBMCs by pH1N1 was greater in individuals with an HAI titer of forty or greater than in those with an HAI of less than forty (n = 40 per group).

### 3.2 Responses of M-CD4^+^T cell in tonsillar TMCs to pH1N1/sH1N1 HAs

**Fig. 2:**
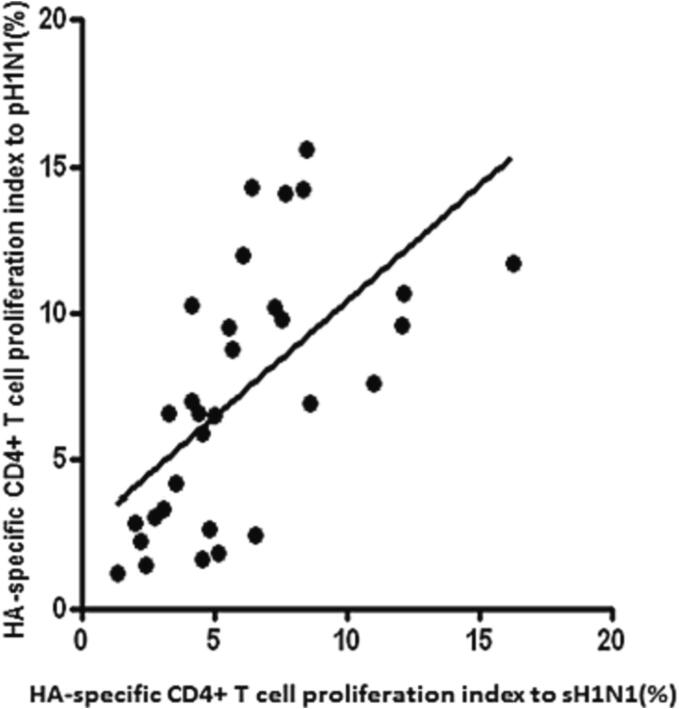
M-C**D4^+^ T cells response to H1N1 HAs.** A robust correlation (r = 0.70) was observed in proliferation indices of M-CD4^+^ T cells induced by exposure to sH1N1 and exposure to pH1N1 HAs (p < 0.01, n = 40).

The relationships of M-CD4^+^ T cell response to different H1N1 HAs after TMCs stimulation was investigated. A robust correlation (r = 0.70) was observed in the proliferation indices of M-CD4^+^ T cells induced by exposure to sH1N1 and exposure to pH1N1 HAs (Fig. 2, p < 0.01).

### 3.3 Impact of subjects’ age on M-CD4^+^ T cell responses to pH1N1

**Fig. 3:**
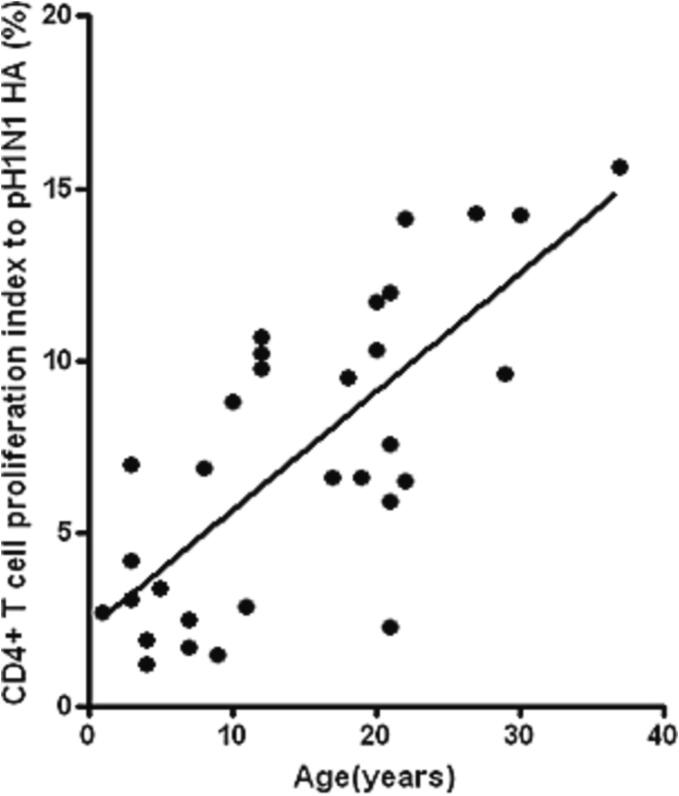
**Impact of subjects’ age on** M-C**D4**^+^**T cells responses to pH1N1.** Subjects’ age correlated ((r = 0.67) positively with M-CD4^+^ T cells responses to pH1N1 (p < 0.01, n = 40).

A positive correlation was found between subjects’ age and M-CD4^+^ T cells responses to pH1N1 (Fig. 3, r = 0.67, p < 0.01). This observation could indicate the gradual development of a cross-reactive anti-influenza memory T cells response. The CD4^+^ T cells response was higher in elderly patients compared to young patients.

### 3.4 Responses of N-CD4^+^ T to pH1N1 in tonsillar TMCs

**Fig. 4:**
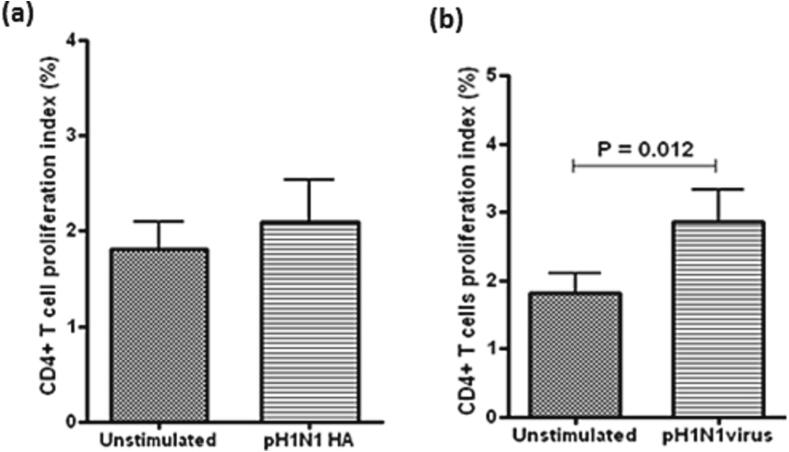
**Responses of TMCs N-CD4**^+^**T cells to pH1N1.** Proliferative index of N-CD4^+^ T cells exposed to pH1N1 HA (a), or to the entire H1N1 virus (b). Significant changes in the proliferative index were induced by the entire virus (b) but not by pN1H1HA protein (a). Paired *t*-test (n = 40).

N-CD4^+^ T cells were exposed to pH1N1 antigen or the entire virus. The proliferation index of N-CD4^+^ T cells was not significantly altered by pH1N1 HA stimulation (Fig. 4**a**, p > 0.05). On the contrary, the proliferation index of N-CD4^+^ T cells was increased after exposure to the entire virus (Fig. 4**b**, p < 0.05). This indicates that the whole virus antigen was immunogenic to stimulate N-CD4^+^ T cells but the purified pH1N1 HA protein was not.

### 3.5 Responses of M-CD8^+^ T cells to pH1N1 in tonsillar TMCs

**Fig. 5:**
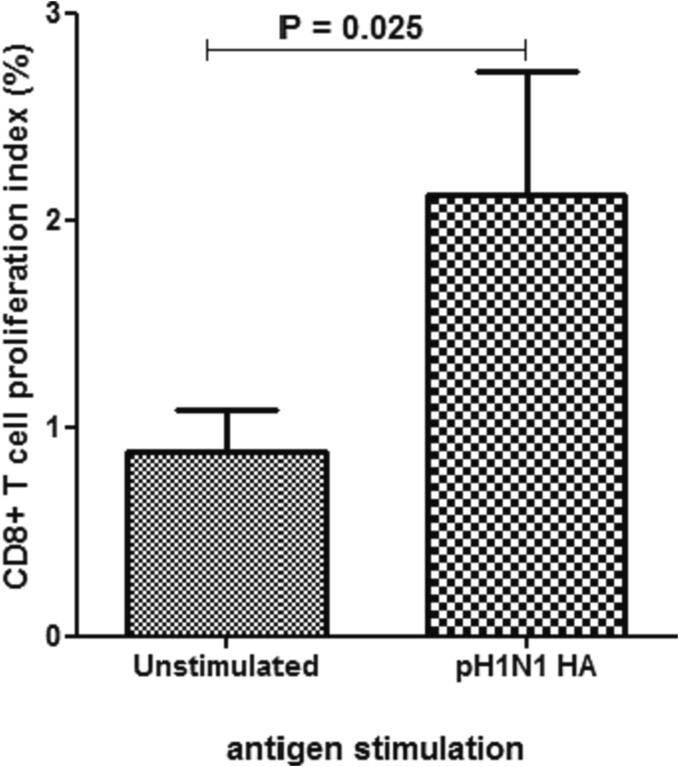
M-C**D8**^+^**T cells responses to pH1N1 in tonsillar TMCs.** M-CD8^+^ T cells proliferation was observed after exposure to pH1N1 (n = 40).

M-CD8^+^ T cells responses to pH1N1 in tonsillar TMCs were examined using the CFSE assay. Exposure of M-CD8^+^ T cells to pH1N1 resulted in a significant elevation of the proliferation index (Fig. 5). Nevertheless, the response of M-CD8^+^ T cells was lower compared to that observed with M-CD4^+^ T cells. Thus, M-CD4^+^ T cells seems to be more responsive to pN1H1 than M-CD8^+^ T cells.

## 4 Discussion

Influenza prevention is the main goal of immunization. Not much is known about cellular immunity in the presence of influenza antibodies in our bodies. Immunity to influenza infection is generally associated with T cell populations (Jiang et al., 2023, Mettelman et al., 2023, Schmidt and Lapuente, 2021).

Tonsils contain an enormous amount of mucosal immune cells including lymphocytes that play an important role against respiratory pathogens (Russell & Mestecky, 2022). Tonsils also serve as a reservoir of immune memory cells that contribute to respiratory tract's defense and provide the benefits of vaccination and protection from infection (Matsuda et al., 2021, Wagar et al., 2021). For example, evaluating B cell responses to intranasal influenza vaccines is a viable approach and has been recently investigated (Mahallawi & Zhang, 2023).

In TMCs, significant M-CD4^+^ T cells responses to pH1N1 were observed. It was hypothesized that after the H1N1 pandemic in 2009, individuals developed M-CD4^+^ T cells responses to pH1N1. PBMCs also harbor M-CD4^+^ T cells responses to pH1N1 virus. However, the latter was moderate compared to TMCs. This discovery is supported by the finding that M-CD4^+^ T cells is prevalent throughout the body (Künzli & Masopust, 2023). Nevertheless, the focus has been primarily on mucosal sites (Sathaliyawala et al., 2013).

The results found that M-CD4^+^ T cells responses to sH1N1 and pH1N1 were related. This provides evidence that common epitopes that stimulate M-CD4^+^ T cells are present in the HAs of both strains. The phenomenon of cross-reactivity has been reported in previous studies (Jing et al., 2016, Namuniina et al., 2023, Pothast et al., 2022). These results are in agreement with a study that reported cross-reactivity in memory B cell responses to H1N1 antigens that mainly relies on M-CD4^+^ T cells (Mahallawi et al., 2013, Mahallawi and Zhang, 2023). Cross-reactivity of M-CD4^+^ T cells to sH1N1 and pH1N1 has been previously reported (Wilkinson et al., 2012). Long-term M-CD4^+^ T cells has been shown to be induced by vaccination and natural infections with determinants in responses showing both seasonal and pandemic genotypes (Teijaro et al., 2010).

A decreased risk of influenza infection has been shown to be associated with individuals with a HAI titer of ≥ 40 (Clark et al., 2017, Kaufmann et al., 2017). In the current study, individuals with at least 40 anti-pH1N1 HAI exhibited a robust M-CD4^+^ T cells response to pH1N1. The pH1N1 presumably induced the HA M-CD4^+^ T cells response assuming prior re-exposure to sH1N1 and cross-reactivity of M-CD4^+^ T cells to sH1N1/pH1N1 (Alam & Sant, 2011). Combining these factors has been suggested to stimulate this heterosubtypic immunity (Pica et al., 2012, Wrammert et al., 2011).

The results showed age-dependent responses of M-CD4^+^ T cells to pH1N1. This finding agrees with a study that reported variations in the frequency of M-CD4^+^ T cells between different age groups (Sathaliyawala et al., 2013).

A moderate response to pH1N1 of M-CD8^+^ T cells was detected compared to M-CD4^+^ T cells. This finding agrees with a study that the response to HA was mostly M-CD4^+^ T cells dependent (L. Y. Lee et al., 2008). This indicates that M-CD8^+^ T cells may be less common in NALT than M-CD4^+^ T cells. Alternatively, different immune cell types may be selective for a distinct category of influenza proteins (Fonteneau et al., 2003).

In summary, pH1N1 HA elicits a substantial M-CD4^+^ T cells response in TMCs. Additionally, this response correlates with the response to sH1N1 suggesting cross-reactivity in T epitopes directed against HAs of both viral strains.

**Funding**

The authors thank “Deputyship for Research and Innovation/Ministry of Education”, Saudi Arabia for funding the study under the project number (445–9-778).

## Declaration of competing interest

The authors declare that they have no known competing financial interests or personal relationships that could have appeared to influence the work reported in this paper.

## References

[b0005] Alam S., Sant A.J. (2011). Infection with seasonal influenza virus elicits CD4 T cells specific for genetically conserved epitopes that can be rapidly mobilized for protective immunity to pandemic H1N1 influenza virus. J. Virol..

[b0010] Aljeraisi T.M., Alomar S.Y., Mahallawi W.H. (2023). BCG vaccine-induced mucosal humoral immunity in human Nasal Associated Lymphoid Tissue. J. King Saud Univ.-Sci..

[b0015] Altorki T.A., Abdulal R.H., Suliman B.A., Aljeraisi T.M., Alsharef A., Abdulaal W.H., Hashem A.M. (2023). Robust memory humoral immune response to SARS-CoV-2 in the tonsils of adults and children. Front. Immunol..

[b0020] Casadei E., Salinas I. (2019). Comparative models for human nasal infections and immunity. Dev. Comp. Immunol..

[b0025] Clark A.M., DeDiego M.L., Anderson C.S., Wang J., Yang H., Nogales A., Topham D.J. (2017). Antigenicity of the 2015–2016 seasonal H1N1 human influenza virus HA and NA proteins. PLoS One.

[b0030] Dotiwala F., Upadhyay A.K. (2023). Next generation mucosal vaccine strategy for respiratory pathogens. Vaccines (basel).

[b0035] Eriksson J.C., Davidsson A., Garberg H., Brokstad K.A. (2003). Lymphocyte distribution in the tonsils prior to and after influenza vaccination. Vaccine.

[b0040] Fonteneau J.F., Gilliet M., Larsson M., Dasilva I., Münz C., Liu Y.J., Bhardwaj N. (2003). Activation of influenza virus-specific CD4+ and CD8+ T cells: a new role for plasmacytoid dendritic cells in adaptive immunity. Blood.

[b0045] Hunt A.M., Shallenberger W., Ten Eyck S.P., Craig F.E. (2016). Use of internal control T-cell populations in the flow cytometric evaluation for T-cell neoplasms. Cytometry B Clin. Cytom..

[b0050] Jiang N., Malone M., Chizari S. (2023). Antigen-specific and cross-reactive T cells in protection and disease. Immunol. Rev..

[b0055] Jing L., Laing K.J., Dong L., Russell R.M., Barlow R.S., Haas J.G., Koelle D.M. (2016). Extensive CD4 and CD8 T Cell Cross-Reactivity between Alphaherpesviruses. J. Immunol..

[b0060] Kaufmann L., Syedbasha M., Vogt D., Hollenstein Y., Hartmann J., Linnik J.E., Egli A. (2017). An Optimized Hemagglutination Inhibition (HI) Assay to Quantify Influenza-specific Antibody Titers. *J vis Exp*(130).

[b0065] Künzli M., Masopust D. (2023). CD4(+) T cell memory. Nat Immunol.

[b0070] Kurono Y. (2022). The mucosal immune system of the upper respiratory tract and recent progress in mucosal vaccines. Auris Nasus Larynx.

[b0075] Lartey S., Zhou F., Brokstad K.A., Mohn K.G., Slettevoll S.A., Pathirana R.D., Cox R.J. (2020). Live-Attenuated Influenza Vaccine Induces Tonsillar Follicular T Helper Cell Responses That Correlate With Antibody Induction. J Infect Dis.

[b0080] Lee, L. Y., Ha do, L. A., Simmons, C., de Jong, M. D., Chau, N. V., Schumacher, R., . . . Dong, T. (2008). Memory T cells established by seasonal human influenza A infection cross-react with avian influenza A (H5N1) in healthy individuals. *J Clin Invest, 118*(10), 3478-3490. doi:10.1172/jci32460. 10.1172/JCI32460 PMC2542885 18802496

[b0085] Lee B.O., Rangel-Moreno J., Moyron-Quiroz J.E., Hartson L., Makris M., Sprague F., Randall T.D. (2005). CD4 T cell-independent antibody response promotes resolution of primary influenza infection and helps to prevent reinfection. J Immunol.

[b0090] Mahallawi W.H., Aljeraisi T.M. (2021). In vitro cell culture model of human nasal-associated lymphoid tissue (NALT) to evaluate the humoral immune response to SARS-CoV-2 spike proteins. Saudi J Biol Sci.

[b0095] Mahallawi W.H., Aljeraisi T.M. (2021). Infection with SARS-CoV-2 primes immunological memory in human nasal-associated lymphoid tissue. Clin Immunol.

[b0100] Mahallawi W.H., Zhang Q. (2023). Live attenuated influenza vaccine induces broadly cross-reactive mucosal antibody responses to different influenza strains in tonsils. Saudi J Biol Sci.

[b0105] Mahallawi W.H., Kasbekar A.V., McCormick M.S., Hoschler K., Temperton N., Leong S.C., Zhang Q. (2013). Infection with 2009 H1N1 influenza virus primes for immunological memory in human nose-associated lymphoid tissue, offering cross-reactive immunity to H1N1 and avian H5N1 viruses. J Virol.

[b0110] Matsuda K., Migueles S.A., Huang J., Bolkhovitinov L., Stuccio S., Griesman T., Connors M. (2021). A replication-competent adenovirus-vectored influenza vaccine induces durable systemic and mucosal immunity. J Clin Invest.

[b0115] Mettelman R.C., Souquette A., Van de Velde L.A., Vegesana K., Allen E.K., Kackos C.M., Thomas P.G. (2023). Baseline innate and T cell populations are correlates of protection against symptomatic influenza virus infection independent of serology. Nat Immunol.

[b0120] Namuniina, A., Muyanja, E. S., Biribawa, V. M., Okech, B. A., Ssemaganda, A., Price, M. A., . . . Redd, A. D. (2023). High proportion of Ugandans with pre-pandemic SARS-CoV-2 cross-reactive CD4+ and CD8+ T-cell responses. *medRxiv*. doi:10.1101/2023.01.16.23284626. 10.1371/journal.pgph.0001566 PMC10431628 37585383

[b0125] Pacini M.F., Balbi C.B., Dinatale B., González F.B., Prochetto E., De Hernández M.A., Pérez A.R. (2023). Intranasal trans-sialidase-based vaccine against Trypanosoma cruzi triggers a mixed cytokine profile in the nasopharynx-associated lymphoid tissue and confers local and systemic immunogenicity. Acta Trop.

[b0130] Passàli, D., Damiani, V., Passàli, G. C., Passàli, F. M., & Bellussi, L. (2003). *Recurrent and chronic inflammations of Waldeyer's ring in childhood: infectious, structural and immunological features.* Paper presented at the International Congress Series.

[b0135] Pica N., Hai R., Krammer F., Wang T.T., Maamary J., Eggink D., Palese P. (2012). Hemagglutinin stalk antibodies elicited by the 2009 pandemic influenza virus as a mechanism for the extinction of seasonal H1N1 viruses. Proc Natl Acad Sci U S A.

[b0140] Pothast C.R., Dijkland R.C., Thaler M., Hagedoorn R.S., Kester M.G.D., Wouters A.K., Heemskerk M.H.M. (2022). SARS-CoV-2-specific CD4(+) and CD8(+) T cell responses can originate from cross-reactive CMV-specific T cells. Elife.

[b0145] Russell M.W., Mestecky J. (2022). Mucosal immunity: The missing link in comprehending SARS-CoV-2 infection and transmission. Front Immunol.

[b0150] Sandor A.M., Sturdivant M.S., Ting J.P.Y. (2021). Influenza Virus and SARS-CoV-2 Vaccines. J Immunol.

[b0155] Sant A.J., DiPiazza A.T., Nayak J.L., Rattan A., Richards K.A. (2018). CD4 T cells in protection from influenza virus: Viral antigen specificity and functional potential. Immunol Rev.

[b0160] Sathaliyawala T., Kubota M., Yudanin N., Turner D., Camp P., Thome J.J., Farber D.L. (2013). Distribution and compartmentalization of human circulating and tissue-resident memory T cell subsets. Immunity.

[b0165] Schmidt A., Lapuente D. (2021). T Cell Immunity against Influenza: The Long Way from Animal Models Towards a Real-Life Universal Flu Vaccine. Viruses.

[b0170] Sircy, L. M., Ramstead, A. G., Joshi, H., Baessler, A., Mena, I., García-Sastre, A., . . . Scott Hale, J. (2023). Generation of antigen-specific memory CD4 T cells by heterologous immunization enhances the magnitude of the germinal center response upon influenza infection. *bioRxiv*. doi:10.1101/2023.08.29.555253. 10.1371/journal.ppat.1011639 PMC11404825 39283916

[b0175] Teijaro J.R., Verhoeven D., Page C.A., Turner D., Farber D.L. (2010). Memory CD4 T cells direct protective responses to influenza virus in the lungs through helper-independent mechanisms. J Virol.

[b0180] Temperton N.J., Hoschler K., Major D., Nicolson C., Manvell R., Hien V.M., Weiss R.A. (2007). A sensitive retroviral pseudotype assay for influenza H5N1-neutralizing antibodies. Influenza Other Respir Viruses.

[b0185] Valor L., Sarmiento E., Navarro J., Gallego A., Fernandez-Yañez J., Fernandez-Cruz E., Carbone J. (2012). Evaluation of lymphoproliferative responses by carboxy fluorescein succinimidyl ester assay in heart recipients with infections. Transplant Proc.

[b0190] Wagar L.E., Salahudeen A., Constantz C.M., Wendel B.S., Lyons M.M., Mallajosyula V., Davis M.M. (2021). Modeling human adaptive immune responses with tonsil organoids. Nat Med.

[b0195] Wilkinson T.M., Li C.K., Chui C.S., Huang A.K., Perkins M., Liebner J.C., Xu X.N. (2012). Preexisting influenza-specific CD4+ T cells correlate with disease protection against influenza challenge in humans. Nat. Med..

[b0200] Wrammert J., Koutsonanos D., Li G.M., Edupuganti S., Sui J., Morrissey M., Wilson P.C. (2011). Broadly cross-reactive antibodies dominate the human B cell response against 2009 pandemic H1N1 influenza virus infection. J. Exp. Med..

